# Characterizing microscale aluminum composite layer properties on silicon solar cells with hybrid 3D scanning force measurements

**DOI:** 10.1038/srep22752

**Published:** 2016-03-07

**Authors:** Sung-Kuk Bae, Beomjoon Choi, Haseung Chung, Seungwon Shin, Hee-eun Song, Jung Hwan Seo

**Affiliations:** 1 Department of Mechanical Engineering, Hongik University, 94 Wausan-ro, Mapo-gu, Seoul, 121-791, South Korea; 2 Department of Mechanical and System Design Engineering, Hongik University, 94 Wausan-ro, Mapo-gu, Seoul, 121-791, South Korea; 3 Biomedical and Nanoengineering Research Center, Hongik University, 94 Wausan-ro, Mapo-gu, Seoul, 121-791, South Korea; 4 Photovoltaic Research Center, Korea Institute of Energy Research (KIER), 152 Gajeong-ro, Yuseong-gu, Daejeon, 305-343, South Korea

## Abstract

This article presents a novel technique to estimate the mechanical properties of the aluminum composite layer on silicon solar cells by using a hybrid 3-dimensional laser scanning force measurement (3-D LSFM) system. The 3-D LSFM system measures the material properties of sub-layers constituting a solar cell. This measurement is critical for realizing high-efficient ultra-thin solar cells. The screen-printed aluminum layer, which significantly affects the bowing phenomenon, is separated from the complete solar cell by removing the silicon (Si) layer with deep reactive ion etching. An elastic modulus of ~15.1 GPa and a yield strength of ~35.0 MPa for the aluminum (Al) composite layer were obtained by the 3-D LSFM system. In experiments performed for 6-inch Si solar cells, the bowing distances decreased from 12.02 to 1.18 mm while the Si layer thicknesses increased from 90 to 190 μm. These results are in excellent agreement with the theoretical predictions for ultra-thin Si thickness (90 μm) based on the obtained Al composite layer properties.

Solar energy is one of the most promising alternative energy sources for the next generation and has been widely used in a broad range of applications. In order to further popularize photovoltaics, the production cost of photovoltaic (PV) power generation should be reduced to make it as comparably low as that of the existing technologies such as fossil fuels and nuclear power. Previous studies have reported the use of less pure silicon in silicon-based solar cells sharing ~95% of PV markets, which could significantly cut down the production cost of the cell modules^1^,^2^. Accordingly, ultra-thin crystalline silicon (c-Si) solar cells have been proposed and undergone intensive development in recent years^3^,^4^. Although these cells have successfully reduced the power generation cost, the thin c-Si solar cell suffers immensely from the bowing phenomenon, which typically increases along with a decrease in Si wafer thickness. This has occurred in the thermal sintering process between the silicon and the rear contact aluminum (Al) layers due to their different material properties^5^. In particular, several laboratories have experimentally and numerically revealed that the mechanical properties of the sintered Al-Si composite layer deposited on the backside of a PV cell are mainly responsible for solar cell bowing^6^,^7^.

We recently developed a theoretical prediction model of the bow in thin c-Si solar cells by using stress-strain relations among the solar cell sub-layers. This model allowed us to estimate bow distances as well as determine the dominant variables affecting bow phenomenon^8^. We demonstrated that our numerical model was more appropriate for predicting the bow in a wide range of Si layer thicknesses compared to the existing Huster numerical model, which is only applicable in a thicker silicon layer^9^. To our best knowledge, however, the accurate mechanical properties of the sintered Al composite layer on a solar cell have never been explored because the existing measurement systems cannot measure the overall mechanical properties of a membrane layer with a relatively large surface area. In contrast, the mechanical properties of the silicon substrate were investigated decades ago. For this reason, despite their enhanced prediction capability, all the existing numerical models predicting the bow of solar cells have not been widely used in the solar cell research field since these models artificially tune the mechanical property values of the Al layer on a solar cell in order to match the bowing distances obtained experimentally^10^. Consequently, the optimal manufacturing conditions minimizing the bowing for ultra-thin solar cells are still unknown.

In this article, we explore the mechanical properties of the sintered Al layer on solar cells by using our novel hybrid 3-dimensional (3-D) laser scanning force measurement technique. The hybrid 3-D laser scanning force measurement (LSFM) system uses a laser surface scanner integrated with a customized 2-Axis (X–Y) microscale translational stage and a force gauge to track the transient microscale 3-dimensional deformation of the microfabricated Al membrane on which different z-directional forces are continuously applied. By using this system, we are able to determine the elastic modulus and yield strength of the sintered Al layer prepared by removing the silicon layer from the solar cell through the deep reactive ion etching (DRIE) process. The obtained mechanical properties are then validated in comparison with those achieved from additional tests using a nano-indentation method. Through this demonstration, we first aim to show the feasibility of our novel approach for measuring the material properties of sub-layers constituting a solar cell. Hence, the bowing distances along with the c-Si layer thickness changes are measured in the experiments. Finally, with these experimental results, we are able to verify whether our numerical prediction model, to which these property values are applied, is capable of estimating the bow phenomena of c-Si solar cells with more accuracy.

## Methods

### Microfabrication

**Figure 1:**
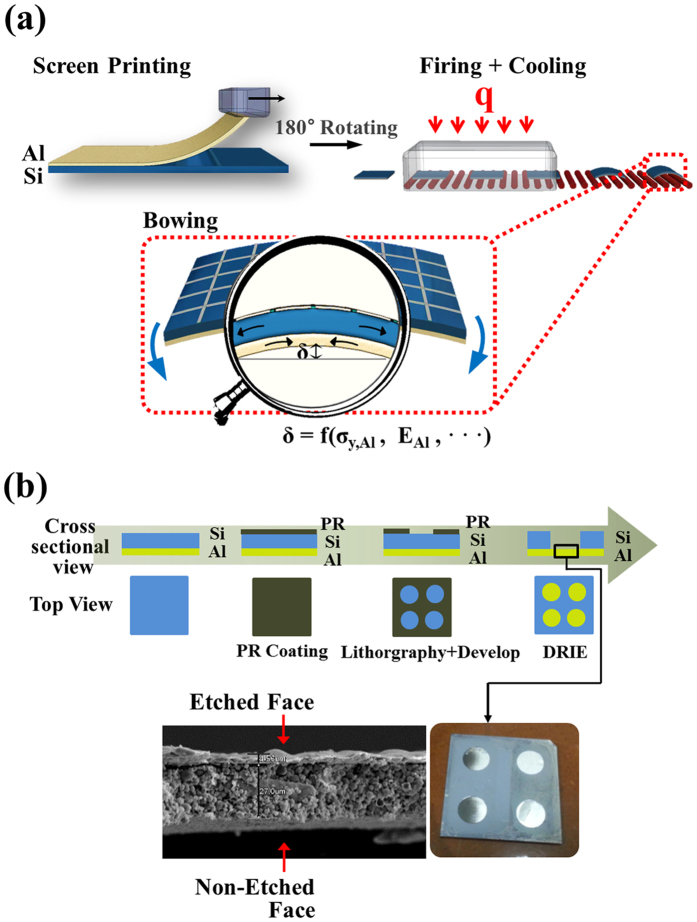
Conceptual illustrations of (**a**) solar cell fabrication and (**b**) micro-fabrication process of the sintered Al layer sample.

In fabricating the solar cell, boron-doped *p*-type crystalline silicon wafers grown by the Czochralski method with 1.1–3.6 Ω∙cm resistivity and 180 μm thickness were utilized. The silicon wafers were textured in the alkaline solution to reduce the reflectance, and then phosphorus was diffused to obtain ~80 Ω/sheet resistance in a POCl_3_ furnace. The industrial rear-etching process was performed to achieve edge junction isolation. The anti-reflection coating (ARC) layers of 80 nm SiN_x_ were deposited by PECVD. The front and rear electrodes were formed by screen-printing with Ag and Al paste, respectively. After drying, both sides of the solar cell were sintered when the cell passed through a belt furnace. Finally, the sintered solar cell was cooled down under room temperature and the bowing suddenly occurred, as shown in Fig. 1a.

To assess the mechanical properties of the sintered rear contact Al composite layer on a solar cell, a thin silicon wafer of 180 μm was textured by wet etching in alkaline solution, followed by the screen printing of Al paste (~30 μm). The Al paste (part no. DSCP A151) was purchased from Dongjin Semichem Co. (Seoul, South Korea). After that, the wafer was sintered in a belt firing furnace (Sierra Therm, 7K15-70C96-5LIR) with multi electrical heating zones (belt width: 380 mm, heating zone length: 1780 mm) without any Ag electrode deposition process. The wafer was then diced. Five different heating zones were specified from 400 °C at the entrance being increased to 870 °C at the final stage. The actual sintering temperature measured on the solar cell surface was 600 °C and the belt translational speed was 0.08 m/s. The experimental condition was set to match the operating conditions used in actual production lines. A new photoresist layer on each diced sample was patterned to expose the sintered Al layer. Through-wafer deep reactive ion etching (DRIE) was used to create a test sample with a circular Al composite membrane (diameter: 1 cm) by removing the silicon layer (Fig. 1b).

### Hybrid 3-D laser scanning force measurement system

**Figure 2:**
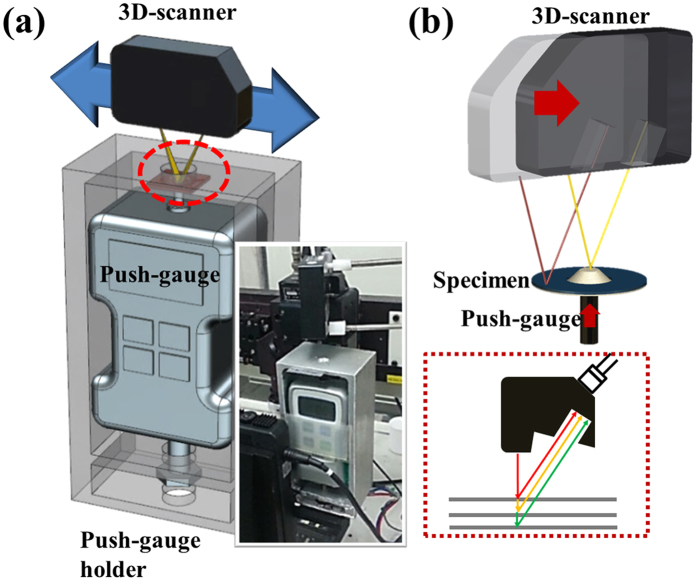
(**a**) Conceptual diagram of 3-D LSFM system. The 3-D LSFM system consists of a 3-D laser scanner, a push-gauge, and a push-gauge holder. (**b**) Schematic of the 3-D laser scanner incorporated into the 3-D LSFM system showing its working principle.

The hybrid 3-D laser scanning force measurement (LSFM) system consists of a microscale translational staged integrated 3-D laser scanner for measuring the transient deformation of a membrane structure, a pedestal for a membrane sample to hold its position while the external force is applied, and a customized force-gauge with a flat tip head diameter of 5 mm for pushing the membrane surface (Fig. 2a). The thin Al composite layer sample sintered at 600 °C is placed on the pedestal integrated with the customized force gauge. Subsequently, the center of the Al layer sample, the force gauge tip, and the laser light of the 3-D laser scanner are precisely aligned. The surface deflection of the Al layer increases proportionally to a vertical force applied to the center of the Al layer by the force-gauge flat tip head, followed by 3-D laser scanning that yields the real-time surface configuration of the Al layer (Fig. 2b). When the Al layer is exposed to the laser light, its reflective angle changes with the Al surface deformation. The reflected light is then redirected into a photo detector integrated with a 665 nm diode laser. By monitoring the reflected light angle shift, the kinetic information of the real-time deformation of the Al surface is acquired. For the pushing load (*P*) uniformly applied along the force-gauge flat tip head of radius *b*, the maximum deflection (*w*) of the Al membrane of radius *a* is estimated as follows^10^:


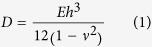



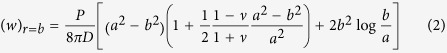


where *D* is the parameter of flexural rigidity that mainly dominates the elastic properties of the thin membrane, while *E*, *h*, and *v* are the Young’s modulus (elastic modulus), thickness, and Poisson’s ratio of the Al layer, respectively. By substituting the pushing load and the measured deflection into eqs (1) and (2), we can obtain the value of Young’s modulus of the Al layer. Also, the yield stress of the Al thin layer is estimated by theoretical analysis using Abaqus (Dassault Systèmes, Vélizy-Villacoublay, France). The parameters essential for the theoretical analysis are determined by measuring the applied pushing load and the corresponding deflection when the membrane fracture occurs. To keep the computational task reasonable, the simulated membrane transition geometries under several different pushing load conditions are examined experimentally.

### Nano-indentation testing

**Figure 3:**
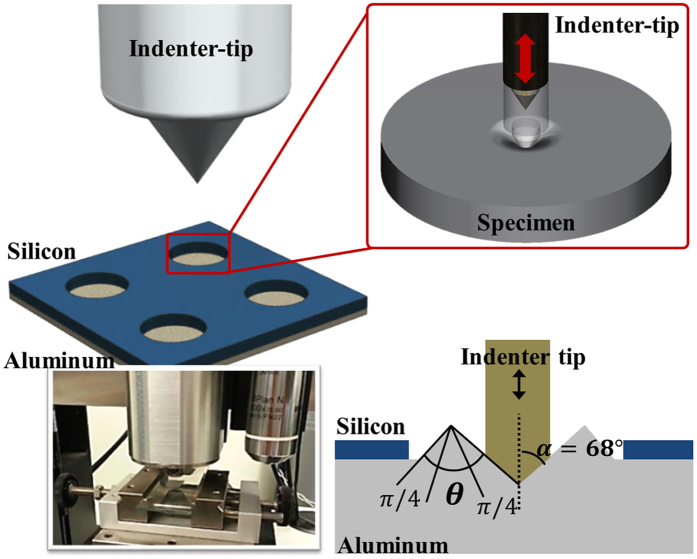
(**a**) Exploded view conceptual diagram of a nano-indenter. (**b**) Image of the nano-indenter used to measure the elastic modulus and the yield stress of the Al membrane. (**c**) Diagram illustrating the concept of the nano-indentation method to estimate the mechanical properties of the Al layer.

As part of a comparative assessment of the mechanical properties of the sintered Al layer obtained from the hybrid 3-D LSFM system, a nano-indenter (NHT-CPX, CSM Instruments SA, Switzerland) specifically designed for mechanical characterization testing is used (Fig. 3b). As shown in Fig. 3a, an indenter tip, normalto the Al sample surface placed on a pedestal of the nano-indenter, is driven into the sample by applying an increasing load up to a pre-determined value. The nano-indenter then continuously monitors both the Vickers hardness as a function of shear strength (*τ*_0_) and the penetration depth of the indenter tip as a function of load during the indentation test. From the information of the Vickers hardness (*H*_*v*_), the indenter tip angle (*α*), and the surface morphology of the deformed material, the yield stress can be determined on the basis of the unified tensile fracture criterion (UTFC) proposed by Zhang and Eckert^11^. The elastic modulus of the Al layer can be easily determined by the relationship between the depth and forces involved during indentation tests. Assuming the material is non-porous, the yield stress could be expressed as follows:


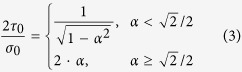


where 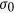 is the yield stress of a non-porous material. However, the deduction in yield criterion for a porous material should be considered since the sintered Al membrane sample, which is similar to ceramics, consists of porous materials^12^,^13^. Thus, the yield stress of the Al membrane sample can be expressed as:


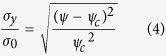


where 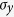 is the yield stress of the Al membrane sample, *ψ* is the material porosity, and *ψ*_*c*_ is the critical porosity at which the yield stress of the material becomes zero. In addition, the elastic modulus of the Al membrane samples could be easily determined using Sneddon’s elastic solution, which is generally used and embedded in the nano-indenter^14^.

## Results

### Elastic modulus and yield stress of the sintered Al layer

**Figure 4:**
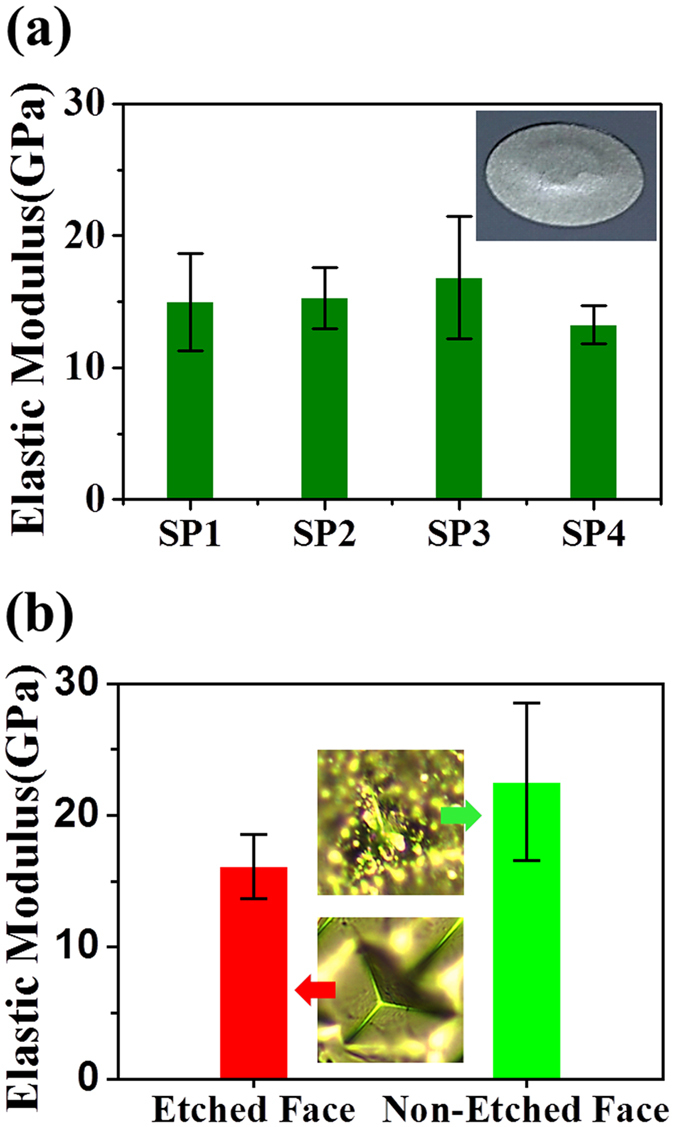
Plots of the elastic modulus of the Al layer measured by (**a**) the 3-D LSFM system and (**b**) the nano-indenter.

In order to assess the elastic modulus of the Al composite layer with the hybrid 3-D LSFM, Poisson’s ratio of 0.326, an essential parameter in eqs (1) and (2), was first experimentally determined using a micro-tensile tester. All the Al composite layer samples tested here were sintered at 600 °C in the furnace because it was very difficult to precisely control the sintering temperature with the current furnace specifications. Figure 4a shows the elastic modulus obtained using the hybrid 3-D LSFM. The elastic modulus of 15.0 GPa, 15.3 GPa, 16.8 GPa, and 13.3 GPa were obtained from the hybrid 3-D LSFM for the 4 individual Al layer samples, respectively. All the samples were tested under 5 different pushing load conditions. The relative standard deviation (RSD) was ≤25% for all cases. These experimental results yield a mean elastic modulus of ~15.1 GPa for the sintered Al layer with discrepancies ranging from 0.5 GPa (corresponding to 1% of the mean elastic modulus) to 1.84 GPa (12.2% of the mean elastic modulus) between the average value and the experimental data. On the other hand, as shown in Fig. 4b, an elastic modulus of 16.1 GPa (Number of tests: 13, RSD: 15%) and 22.6 GPa (Number of tests: 9, RSD: 25%) were obtained from the nano-indentation testing for the etched face by DRIE and the non-etched face of the Al layer sample, respectively. The relatively large RSDs might have been caused by the surface non-uniformity due to the material porosity.

**Figure 5:**
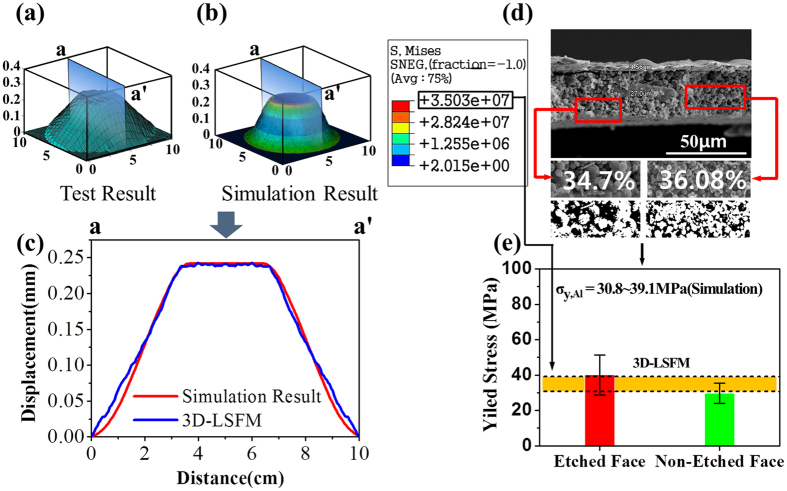
(**a**) Scanned 3-D surface configuration of the Al layer at a pushing load of 0.74 N just before plastic deformation occurs. (**b**) Finite element simulation of the Al layer deformation for the corresponding load condition. (**c**) Modeled (red line) and experimental (blue line) surface displacement under a pushing load of 0.74 N by the 3-D LSFM system. (**d**) SEM image of the cross-sectional Al layer, which is capable of measuring the material porosity. (**e**) Plot of yield stress for the etched (red) and non-etched (green) faces using the nano-indenter. The 3-D LSFM test result is also plotted as a yellow area for comparison.

Subsequent tests performed with the 3-D LSFM afforded more accurate and precise estimates of the yield stress. Figure 5a shows the scanned surface shape of an Al layer sample at a maximum pushing load of 0.74 N just before plastic deformation occurs. The maximum loads for 4 different samples without plastic deformations were 0.74 N, 0.74 N, 0.60 N, and 0.43 N, respectively. The discrepancies in the maximum pushing loads were caused by morphological surface non-uniformity for each of the samples. For the corresponding load conditions, an average yield stress of 35.0 MPa was estimated using Abaqus analysis that considered the Al layer sample geometries and the experimentally obtained elastic modulus (Fig. 5b). Figure 5c shows that the predicted cross-sectional surface configurations agree with the experimental results with a ±1% error. For the yield stress tests with a nano-indenter, the porosity of the Al layer was measured with ImageJ on the basis of the cross-sectional SEM image of the Al layer. An average porosity of 36.1% was obtained from 5 different regions on the cross section of the Al layer with an RSD <5% in all cases (Fig. 5d). From the aforementioned UTFC that considered the deduction in yield criterion due to material porosity, we achieved a yield stress of 29.7 MPa (RSD: 19.0%) and 40.1 MPa (RSD: 28.1%) for the etched and non-etched faces, respectively. These yield stress values were 15.1% lower and 14.6% higher than the mean value of 35.0 MPa achieved by the 3-D LSFM tests. For each of the 4 individual Al membrane samples, yield stresses ranging from 30.8 MPa to 39.1 MPa were obtained using our 3-D LSFM system. This result indicates that the porosity variations together with the surface non-uniformities more significantly amplify the errors in the yield stress calculation for the use of the nano-indenter.

### Effect of mechanical properties of the sintered Al layer on the bowing phenomena of c-Si solar cells

**Figure 6:**
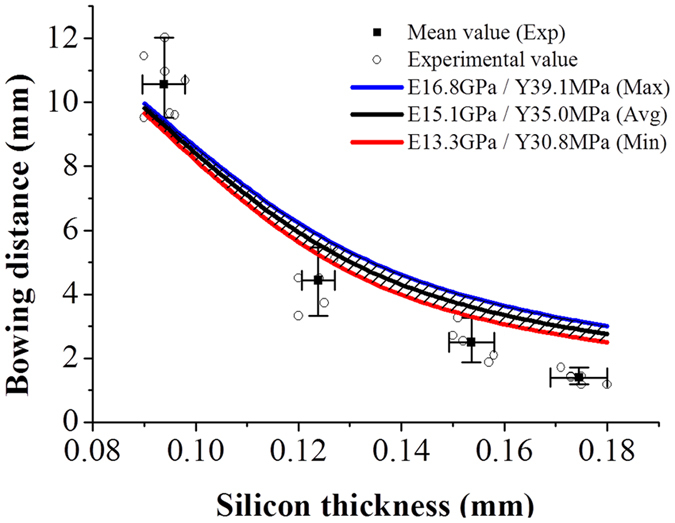
Modeled (3 lines (red, black, blue)) and experimental (circles) values of bowing distance versus the silicon substrate thickness of a Si solar cell.

Our previous study^1^,^8^ indicated that a higher elastic modulus and lower yield strength of the sintered Al layer can yield a smaller bowing distance. A high elastic modulus with a low yield point for the Al layer is expected to provide a minimized bowing phenomenon. However, our numerical bowing prediction model was developed on the basis of an inaccurate elastic modulus and yield strength of the Al layer, i.e., the yield strength value of 15~36 MPa recommended by Huster^9^ and the elastic modulus value of 3.5~31 MPa chosen by Chen^15^ and Popovich^16^. Thus, we re-performed the numerical analysis by using our model based on the actual Al layer properties (i.e., yield strength: 35.0 MPa, elastic modulus: 15.1 GPa) obtained from the 3-D LSFM, followed by comparingthe modeled bowing distances with those measured experimentally. The experimental results plotted in Fig. 6 show that the bowing distance increased exponentially along with a decrease in the silicon layer thickness at a modest rate from 180 μm to 120 μm and at an apparently higher rate below 100 μm. As shown by the 3 lines for the 3 modulus conditions (red: minimum elastic modulus, black: average elastic modulus, blue: maximum elastic modulus) in Fig. 6, the agreement of the model with the experimental results is reasonably excellent, particularly with the extremely thin c-Si solar cell tested. The experimental bowing distance value of 10.56 mm at the thinnest Si thickness of 90 μm is only 7% higher than that predicted by the model. For the numerical analysis, the relative standard deviation in the bowing distances was ≤1.5% below the Si layer thickness of 120 μm and ≤9.1% for the rest of the cases. Notably, compared to the other existing prediction models, our model provides more accurate estimation of the bowing distance in the thinner range of c-Si solar cells.

## Discussion

This is the first report that examined both the elastic modulus and yield stress of a microscale Al layer sintered on silicon solar cells using a hybrid 3-D LSFM system and a nano-indenter. The 3-D LSFM has a force gauge integrated with a 3-dimensional laser surface scanner that permits *in situ* surface configuration of a deformed membrane layer under a loading force in the center. The results presented demonstrate that the 3-D LSFM system has a unique ability to observe more precise and consistent overall mechanical properties of sub-layers constituting a solar cell regardless of those material porosities and surface non-uniformities. These unique features constitute a significant advantage over the nano-indentation method, which is remarkably sensitive to local surface non-uniformities. With this observation, we obtained an elastic modulus of 15.1 GPa and a yield strength of 33.0 MPa for the Al layer. Furthermore, we re-evaluated our numerical bowing prediction model by applying the obtained mechanical property values of the Al layer. The model predicted the bowing distances of each Si layer thickness of solar cells, which are in remarkably good agreement with the experimental results, particularly at an ultra-thin c-Si solar cell of ≤100 μm, whereas the bowing distances were overestimated in the thicker range.

Ongoing work is focusing on more in-depth studies of building the material property library of solar cell sub-layers, thus providing optimal manufacturing conditions to mitigate the bowing phenomena of solar cells. Although we confined our 3-D LSFM for the analysis of the solar cell bowing due to the Al layer, the 3-D LSFM could be applied to any thin-film based multi-layer systems when the targeted membrane layers are prepared by proper microfabrication techniques. Together with this modification, our prediction model presented here opens the way for our future development of ultra-thin c-Si solar cells by allowing for precise estimation of the bowing phenomenon under a variety of different design conditions. In this paper, we have taken a significant step towards the realization of ultra-thin solar cells by providing the numerical bowing prediction model as well as experimentally characterizing the Al composite layer of solar cells using the 3-D LSFM.

## Additional Information

**How to cite this article**: Bae, S.-K. *et al.* Characterizing microscale aluminum composite layer properties on silicon solar cells with hybrid 3D scanning force measurements. *Sci. Rep.* **6**, 22752; doi: 10.1038/srep22752 (2016).
